# Modeling Radio Wave Propagation for Wireless Sensor Networks in Vegetated Environments: A Systematic Literature Review

**DOI:** 10.3390/s22145285

**Published:** 2022-07-15

**Authors:** Alexis Barrios-Ulloa, Paola Patricia Ariza-Colpas, Hernando Sánchez-Moreno, Alejandra Paola Quintero-Linero, Emiro De la Hoz-Franco

**Affiliations:** 1 Department of Computer Science and Electronics, Faculty of Engineering, Universidad de la Costa, Barranquilla 080002, Colombia; pariza1@cuc.edu.co; 2 Department of Electronics Engineering, Faculty of Engineering, Universidad de Sucre, Sincelejo 700001, Colombia; 3 Facultad de Ciencias Básicas, Universidad Simón Bolívar, Centro de Investigación e Innovación en CienciasMarinas y Limnológicas del Caribe Colombiano “CICMAR”, Barranquilla 080001, Colombia; hsanchez13@unisimonbolivar.edu.co; 4 Microbiology Program, Universidad Popular del Cesar, Valledupar 200002, Colombia; alejandraquinterol@unicesar.edu.co

**Keywords:** attenuation, vegetated environments, propagation models, path loss, systematic revision of literature, wireless technologies, WSN

## Abstract

The use of wireless sensor networks (WSN) for monitoring variables in agricultural environments and natural forests has been increasing in recent years. However, the sizing of these systems is affected by the inaccuracy of the radio wave propagation models used, leading to possible increased costs and measurement errors. This systematic literature review (SLR) aims to identify propagation models widely used in WSN deployments in agricultural or naturally vegetated environments and their effectiveness in estimating signal losses. We also identified today’s wireless technologies most used in precision agriculture (PA) system implementations. In addition, the results of studies focused on the development of new propagation models for different environments are evaluated. Scientific and technical analysis is presented based on articles consulted in different specialized databases, which were selected according to different combinations of criteria. The results show that, in most of the application cases, vegetative models present high error values when estimating attenuation.

## 1. Introduction

Wireless sensor networks (WSNs) based on the Internet of Things (IoT) are widely used in different scenarios, including agricultural or with the presence of natural vegetation. These tools are used to obtain the values of the different variables of a plantation or forest, whether related to climate, soil, or plants. They are one of the most promising data collection methods in agricultural applications due to their low cost and improvements in environmental monitoring [1,2]. This information is useful for the personnel in charge of crop management as it facilitates the management of the processes in their plantations [3,4]. This form of crop management is called precision agriculture (PA) and allows managers to more efficiently manage both the facilities and the inputs required by the plantation. It is important to highlight that the monitoring of variables in PA contexts has a positive impact on irrigation management, reduction in environmental impact, and fertilizer management, among others [5,6]. 

The number of nodes that can be deployed and interconnected ranges from one or two units to thousands, depending on geographical coverage and topography [5], which is enhanced by the mMTC (Massive Machine-Type Communication) scenario of 5G [7,8]. The correct deployment of the nodes in the field is based on the efficient management of the transmitter power, knowing beforehand the signal attenuation through an adequate propagation model that leads to adequate data collection [9,10,11].

**Figure 1:**
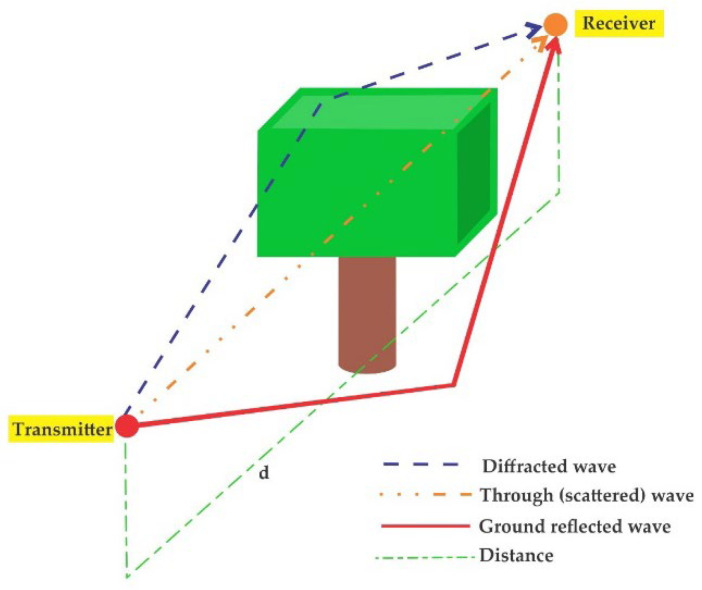
Examples of propagation modes in vegetation. Source: Refs. [17,18].

The wireless signal is often obstructed in its path by trees, hills, mountains, and buildings, among others [12,13], resulting in phenomena such as scattering, diffraction, and reflection [14] (see Figure 1). Even the signal strength in line-of-sight (LOS) links and static transceivers can be altered due to scatterers or moving objects affecting the propagation environment [15]. For this reason and given the importance of WSN implementation in PA contexts, it is necessary to bring propagation model estimates closer to observed values as they are closely related to WSN performance prediction metrics (energy loss, path optimization, and link reliability) [15,16].

Propagation models that estimate path loss or characterize attenuation in wireless links [19] play an important role in the deployment of WSNs in different large-scale contexts [20]. As for its application in PA, it is used to model the attenuation through the foliage, estimating metrics useful in planning the deployment of WSNs. In the scientific literature, they are grouped into three main categories: empirical, deterministic, and stochastic [21]. However, it is important to note that this classification cannot be considered accurate as some models can be classified in more than one category without being mutually exclusive. The empirical ones are obtained from rigorous field measurements in addition to the analysis of the statistical characteristics describing the behavior of the signal using an equation [22]. Deterministic ones are based on the study of propagation phenomena, as well as on the knowledge of the environment in which the network is deployed; they can be simple and range from only including the distance separating the nodes to considering more complex factors in its development (e.g., multipath fading) [23]. Finally, stochastics result from the modeling of the environment based on random variables [24], which generates high error values in the results.

There are different propagation models oriented to WSNs. However, many of them have high error values or are not very rigorous because they do not consider relevant factors in their equations (e.g., antenna height).

The main objective of this review work is to determine which propagation models are used in the characterization of signal losses of WSNs deployed in agricultural or vegetation environments, as well as their efficiency in different scenarios (forest, outdoor crop, greenhouse, meadow). The specific objectives of this systematic literature review (SLR) are:

- Contextualize the use of propagation models used in WSN planning in PA and vegetated environments.
- Identify applications in vegetation scenarios where propagation studies associated with WSN deployment have been conducted.
- Identify the wireless technologies most used in propagation studies oriented to the deployment of WSNs in agricultural or vegetation environments.

The main motivation of this study is to present an SLR that identifies the most widely used models with the greatest impact on WSNs in agricultural contexts or environments with similar characteristics.

The contributions of this document are specified below:

- Effectiveness of propagation models in WSN applications related to agriculture or forestry environments.
- Identification of the predominant wireless technologies in propagation studies for sizing WSN nodes in agriculture and similar scenarios.
- Identification of the most used techniques to validate propagation models in vegetated environments.

The structure of this article is as follows: Section 2 presents the related works, Section 3 presents the methodology used during the review, Section 4 describes the scientometric analysis, and Section 5 presents the detailed technical analysis. Section 6 and Section 7 present, respectively, the discussion and the results obtained. Finally, Section 8 and Section 9 present the conclusions, recommendations, and future work.

## 2. Related Works

Regardless of the type or way of obtaining each propagation model (also called path loss models), we must be aware of the particularities of the scenario where the WSN is deployed. Therefore, it is normal to (a) consider the models validated and ready to be applied, (b) adjust these models, and (c) develop a new one that allows to adequately characterize the attenuation.

After searching specialized databases, the number of literature review articles on WSN-oriented radio wave propagation models is minimal, let alone focused on their applications in agricultural contexts. In Ref. [15], they reviewed and compared models, describing their limitations in WSNs in general, without focusing on specific scenarios. In Ref. [25], they evaluated different models frequently used in WSN planning through a simulation platform. They demonstrated the usefulness of modeling the network in vegetation environments because their results significantly influence network performance. In Ref. [24], they studied different path loss prediction methods, not specifically intended for WSNs, and presented a new taxonomy of models based on the similarities and differences between them.

Other works, although not of review, evaluate propagation models intended for WSNs in agricultural or similar scenarios of forests or with the presence of pastures. In Ref. [26], they contrasted several empirical models in different bands and crops, relating received signal strength (*RSSI*) values to path loss data. In Ref. [27], the foliage attenuation model recommended by the International Telecommunication Union (ITU-R), the Modified Exponential Decay model (MED), and Weissberger model were simulated and analyzed to predict the connectivity as well as the maximum coverage of the nodes within the communication path. In Ref. [3], they described the effects of distance, antenna height, and vegetation depth on the models. However, these papers focus on specific issues and do not present a comprehensive systematic review on aspects of vegetation propagation modeling for WSNs, such as: the effectiveness of the most used models, study scenarios, or analysis of validation results.

The initial information search shows that the number of SLR or bibliometric review articles on modeling radio wave propagation in WSNs deployed in vegetated environments (e.g., agricultural fields) is small. On the contrary, numerous reviews are focusing, for example, on sensors [28], optimization models [29], WSN applications in PA [30], and the use of communication technologies from frequency bands, data rate, and transmission range, among other aspects [31]. Therefore, researchers new to propagation modeling in vegetated WSN systems do not have a wide variety of review literature at their disposal and must devote part of their working time to gathering a wealth of information due to many scenarios under study, the available wireless technologies, and the lack of unified methods to validate the results. In that sense, this SLR article will be a valuable tool for consultation and support on the subject, not only for the identification of the most used models, scenarios, and technologies in the research on the modeling of radio signal propagation in agricultural WSN systems or similar but also the evaluation and analysis of the effectiveness of these models from the statistical methods used as a validation tool.

## 3. Methodology

Within the field of research, SLRs have become an important contribution to the scientific community since they are articles that compile, summarize, criticize, and synthesize the information available on a specific topic. This study considers the SLR recommendations presented in Refs. [32,33,34,35]. The structure and methodology of the SLR articles used in Ref. [36] were considered, where they established search criteria based on keywords found in previous reviews and exclusion principles that allowed the information found to be purified. 

In the field of WSNs, they defined in Ref. [5] a set of questions to be solved in the implemented SLR. They performed an information search in specialized databases and created search strings from keywords. The selection criteria considered the period of publication (in years), as well as the selection of articles included in journals indexed in the Science Citation Index (SCI). In Ref. [30], they established an SLR process to identify applications and criteria in PA technology selection. Based on these guiding references, SLRs’ work on radio wave propagation models in agricultural environments was developed in three phases: (1) design of the search string, (2) application of the search string and data collection, and (3) analysis of the results.

In phase 1, a series of questions were posed:

Q1. What are the most used propagation models for sizing WSNs in agriculture?

Q2. In which crops, or scenarios with vegetation, has propagation modeling work been done for WSN?

Q3. What are the communication technologies used as a tool for modeling attenuation in WSNs in agricultural or similar environments?

**Figure 2:**
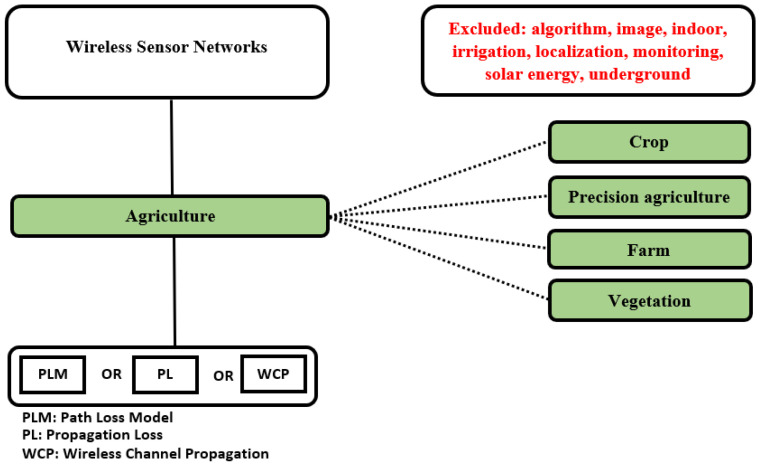
Conceptual map used in the construction of search strings.

Subsequently, the search strings were structured based on the compilation of the information presented in review and overviews articles. Figure 2 shows the conceptual map considered in its construction.

Table 1 presents the thematic axes and explains the strategies for combining these criteria in the elaboration of search strings. The combinations were created in ascending and descending order. In addition, the terms used in the exclusion criteria are presented.

In phase 2, search strings were applied in five highly recognized specialized databases: Scopus, Web of Sciences (WoS), Institute of Electrical and Electronics Engineers (IEEE), Science Direct, and Springer. Other sources of information, such as Google Scholar and ResearchGate, were not considered. On these platforms, there may be cases where researchers link documents that do not correspond to scientific articles. In addition, they do not apply the same rigorous publication standards that apply to specialized databases.

Different terms were used to establish exclusion criteria. The concepts “algorithm”, “image”, “irrigation”, “monitoring”, and “solar energy” were associated with results in which the main topic was the implementation of WSNs for monitoring, so they were not considered. Excluded as well were those articles in which the concepts “indoor” and “localization” could be related to the topic of propagation, but most of them did not contain aspects of WSNs, agriculture, or vegetation. The selected articles were published between 1 January 2011 and 31 December 2021. Those chains that did not yield results, or where the results were not related to this SLR work, they were discarded.

The initial selection process of the articles was based on the analysis of the title, keywords, abstract, and conclusions in order to establish their coherence with the main objective of the research. Other exclusion criteria were language, publication status, and journal classification. Only papers submitted in English were selected, and those in preprint that had not undergone peer review were discarded. In terms of categorization, the selection of journal articles was limited to those ranked between the first and fourth quartile (Q1–Q4) in the Scimago Journal Report (SJR) and the Journal Citation Report (JCR), eliminating uncategorized articles. The latter criterion was not applied to the documents corresponding to the conferences. In the same phase, to strengthen the search, the review included the references presented in the selected papers, adding 14 additional papers. The existence of repeated documents in two or more databases was reviewed in detail, and 69 out of the total number of articles chosen were eliminated and counted only once. 

Initially, 138 articles related to the topic of propagation models for WSNs in agricultural or vegetation environments were selected and analyzed. In this phase, the most relevant references were identified, creating a database with scientific and technical information. Subsequently, a more detailed analysis was carried out, reviewing in each article elements such as problems addressed, methodology used, the technology employed, and problems to be solved, which made it possible to reduce the number of articles to 84. The scientometric and technical variables of each were documented. As an additional criterion for the final selection, the information provided by the authors regarding the measurement or simulation environments, the methodology used, and the presentation of the results was taken into account.

Finally, in phase 3, the results were analyzed based on quantitative and qualitative information, correlating the different categories examined in the review process.

## 4. Scientometric Analysis

**Figure 3:**
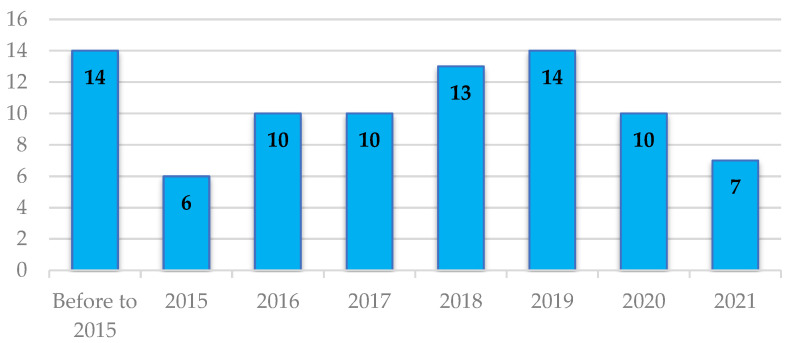
Number of publications per year.

For each of the 84 references, the following scientometric information was quantified and recorded: name of the journal or conference, quartile ranking in Scopus and WoS, nationality of the journal in which it was published, country of origin of the work, and authors’ institution of affiliation. Figure 3 shows the number of articles related to propagation models, WSN, and agriculture.

It is evident that the number of publications on this specific topic demonstrates its relevance within propagation and WSN research, mainly in works related to (1) characterization of attenuation in environments with the presence of vegetation and evaluation of widely recognized models, and (2) development of new propagation models associated with agricultural or vegetated environments. Figure 3 shows that the maximum peak of publications was reached in 2019, before the start of the pandemic caused by the SARS-CoV-2 virus; this situation caused a large part of the research resources to be allocated to the study of aspects related to the pandemic. 

**Figure 4:**
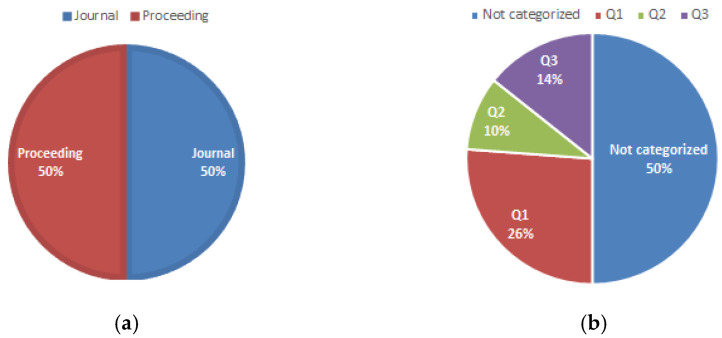
Publications by type and quartile: (**a**) percentage by type of publication and (**b**) percentage of publications by quartile.

Figure 4 shows that 50% of the selected papers were published in journals, while the remaining 50% corresponded to conferences. These indicators, as well as the large percentage of publications, ranked in Q1 (see Figure 4a), demonstrate the relevance of this topic in the field of research.

**Figure 5:**
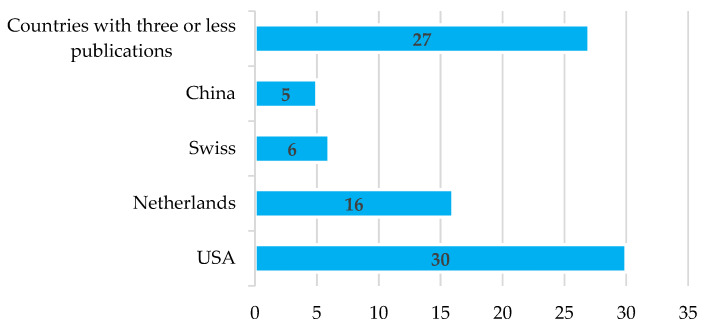
Number of publications received by country.

The large number of countries that have journals or hold conferences dedicated to the subject demonstrates the interest and importance that propagation studies generate within the research community, especially regarding improving the layout of sensor nodes in scenarios where vegetation affects the signal quality of communications links. Figure 5 shows that the countries with the highest number of articles received are the United States, Netherlands, Switzerland, and China, which have five or more publications in their different journals or conference proceedings.

**Figure 6:**
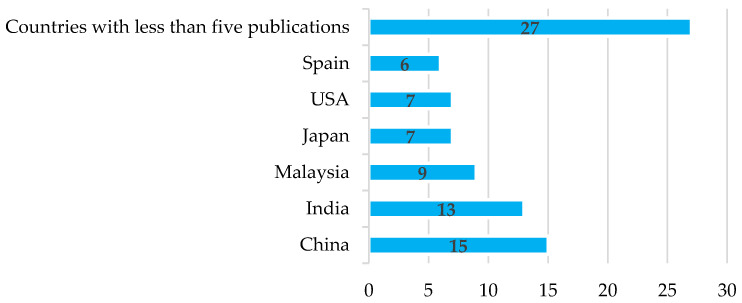
Number of publications generated by country.

Figure 6 presents information on the countries with the largest number of papers related to this SLR. The list is headed by China, followed by India, Malaysia, Japan, the United States, and Spain, nations in which agriculture is of great importance as an economic activity due to their growing population and because they dedicate part of their production to exports. In addition, the information in the figure shows that there is a general interest in much of the world for projects related to research activities in PA, WSN, and propagation. As an evaluation criterion for this item, relevance was given to the country of affiliation of the authors, and, in cases where the origin of more than one country was recorded, the origin of the first author was chosen.

The journals with the highest number of publications related to the topic of this SLR are presented in Table 2. The number of articles, as well as the categorization of the journals according to quartile in SJR and JCR, show that the topic of radio signal propagation aspects in WSNs deployed in agricultural or similar fields is of great importance in the global scientific context.

## 5. Technical Analysis

When examining research that aims to characterize propagation in agricultural or similar environments, different technical aspects are considered. The most relevant considerations resulting from this SLR are presented below from three approaches: radio wave propagation models for WSN in agricultural or vegetated scenarios, application scenarios for propagation studies, and technologies used (wireless, frequency bands, and tools).

### 5.1. Radiowave Propagation Models for WSNs in Agricultural or Vegetated Environments

**Figure 7:**
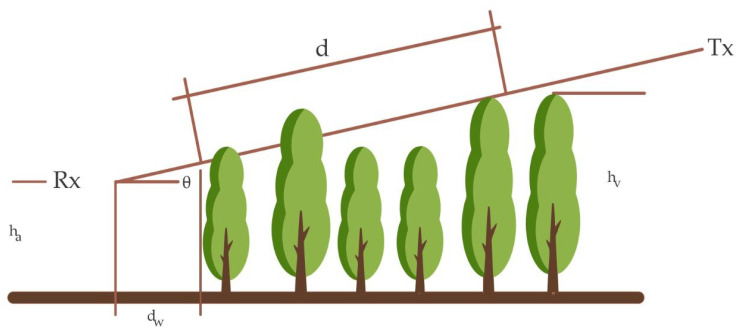
Parameters considered in the propagation path of the radio wave in vegetation. Source: Ref. [37].

The path of radio waves in forest, crops, or similar environments is affected by numerous parameters: the vegetation path length (*d*), the average tree height (*hv*), the height of the receiving antenna (*hr*), the path elevation angle (*θ*), and the distance from the antenna to the road forest (*dw*) [37] (see Figure 7). In addition, each crop and vegetated environment has its propagation characteristics, so many studies aimed at characterizing radio signal attenuation in the wireless channel are based on recognized models. This occurs when the network must be planned and sized, or when the effectiveness of the proposed new models must be evaluated.

**Figure 8:**
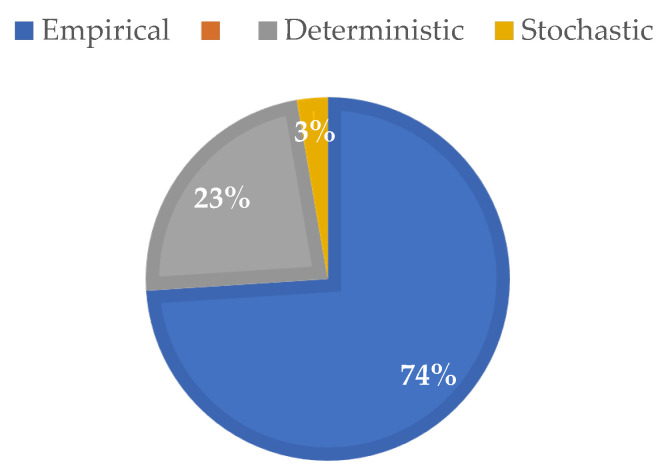
Types of models identified in the SLR.

As a result of this SLR, it became evident that, in agricultural or forestry scenarios, empirical models are the most used in the planning and installation of WSNs, as shown in Figure 8.

Due to their high efficiency in loss estimation, in addition to their low mathematical complexity, empirical models are highly appreciated in wireless network dimensioning. Among them is the MED, one of the most widely used due to the high levels of accuracy it offers when determining canopy losses [38]. The Equation (1) describing it is presented below [39]:

(1) $$Att_{MED}=xf^{y}d^{z}$$

where *f* corresponds to the frequency given in MHz, and *d* is the depth of the vegetation in meters. In addition, *x*, *y*, and *z* are parameters that must be tuned from the measurements made in each scenario where their use is required [40]. In Ref. [27], it was contrasted, along with other models, to establish which one provided greater effectiveness and, thus, adequately sized WSNs in different environments, including agriculture. MED was also applied in Refs. [38,39] for comparison with a model proposed by the authors.

Different models have emerged from the MED, such as Weissberger, ITU-R, adjusted ITU-R (FITU-R), and the COoperation in science and technology (COST-235), which have been widely reported in the literature. In the selected papers in this SLR, the ITU-R model was the most used in the estimation of radio signal power loss in vegetated environments where they implemented or simulated WSNs. The Equation (2) representing the losses of this model is presented below [12]:

(2) $$PL_{ITU-R}\left(\mathrm{dB}\right)=0.2\times f^{0.3}\times d^{0.6}.\;$$

where *f* is the frequency in MHz and *d* corresponds to the distance. The model was used in Ref. [41] in the calculation of signal losses in different types of plantations, in addition to different variations of garden scenarios (coconut trees with green grass, dry open grass, and wet green grass), and, in Ref. [26], a comparison was made concerning the measurements made in each of these scenarios. In Refs. [3,27], they presented models that were examined to determine which best represented the losses in the proposed scenarios. However, in these cases, they were compared with simulations and not with actual measurements. In Refs. [12,18,42,43,44], the ITU-R estimates were compared with the results obtained when developing new models to calculate the effectiveness of the models.

Another widely used model within the set of articles examined in this SLR is COST-235, which also comes from adjustments made to the MED. Equation (3) represents it [45]:

(3) $$C_{L}(\mathrm{dB})=\{\begin{array}{c} 15.6\times f^{-0.009}\times d^{0.26},in\;leaf\\ 26.6\times f^{-0.2}\times d^{0.5},\;out\;of\;leaf \end{array}.\;$$

In this equation, *f* is the frequency value in MHz and *d* is the distance in meters. Like the models described above, COST-235 was also used to identify a suitable method describing foliage losses in crop or forestry scenarios, as is the case reported in Ref. [46], where the impact of the orientation of WSN nodes was also analyzed.

Like ITU-R and COST-235, the Weissberger model is derived from the MED and has been widely used in the work that is part of this SLR. Equation (4) describes the losses of this model [47]:

(4) $$L_{\left(\mathrm{dB}\right)}=\{\begin{array}{c} 1.33\times f^{0.284}\times d^{0.588};si\;14<d\leq 400\\ 0.45\times f^{0.284}\times d;si\;0<d<14 \end{array}.\;$$

In this case, the frequency (*f*) is given in GHz and *d* in meters. This model is only defined at a foliage depth of up to 400 m.

The FITU-R model is also derived from the MED and is one of the most widely used in the articles considered in this work. It was proposed for situations where the transmitting or receiving antenna is located near a small strip of trees so that most of the signal propagates through them. Equation (5) represents it [3]:

(5) $$L_{FITU-R(\mathrm{dB})}=\{\begin{array}{c} 0.37\times f^{-0.18}\times d^{0.54},\;\;out\;of\;leaf\\ 0.39\times f^{-0.39}\times d^{0.25},\;in\;leaf\;\;\;\;\;\;\;\;\;\;\;\;\;\;\;\;\; \end{array}$$

where *f* is expressed in MHz and *d* in meters.

Although empirical models are the most used in the works considered in this review, the use or development of deterministic models is also reported, as is the case of the Two-Ray model. It considers the effects of direct and reflected waves to the ground on signal propagation [48]. Equation (6) describes the losses of this model [49]:

(6) $$P_{LTwo}(\mathrm{dB})=40\log\left(d\right)-20\log\left(h_{t}\right)-20\log\left(h_{r}\right)-10\log\left(G_{t}\right)-10\log\left(G_{r}\right)$$

where *ht* and *hr* are the heights in meters of the transmitting and receiving antennas, respectively, and *Gt* and *Gr* are the gains of the transmitting and receiving antennas in dBi.

Although Two-Ray is one of the most widely used in WSN propagation studies in PA contexts, most papers report its use in comparison to other models to estimate its effectiveness, as presented in Refs. [50,51,52,53]. Furthermore, in Refs. [16,54], it was used in the characterization of WSN radio wave propagation in fields or scenarios with the presence of vegetation. In the SLR performed in Ref. [15], it was considered within the group of most important propagation models in the estimation of signal losses in WSN in agricultural environments or where vegetation is an important factor in the propagation behavior.

Regarding the development of deterministic models, only one case is reported in Ref. [55], where they considered the effects of corn and potato field vegetation on propagation by simulating the dielectric properties of these crops.

Concerning stochastic models, they usually present high error values in the estimation of signal loss in a wireless channel. Despite this, there are reports of papers that considered its use. In Ref. [56], the authors proposed a new model for WSN applied to forest environments using the Green Obadait and other models in evaluating the effectiveness of their proposal. The use of the One-Slope model was also reported in research relating WSNs and PA. In Ref. [57], it was used to validate the use of Xbee modules in agricultural environments; while the SLR presented in Ref. [15] considered it one of the most useful in characterizing signal power loss in WSNs.

Within the wide range of radio wave propagation models that appear in the literature, it is evident that one of the most widely used is the Free Space Loss Model (FSPL). Ideal propagation conditions are assumed here without considering the phenomena of diffraction, reflection, refraction, and absorption. Moreover, it is only valid in the far-field region [58]. However, although it is a general-purpose model, it is very useful in studies of radio signal wave propagation in agricultural plantations or vegetated environments, and as with the Two-Ray, it serves as a complement to the MED or its derivations, such as ITU-R, FITU-R, and COST-235, because the losses predicted by these models are concentrated in the foliage and not in the entire path. Therefore, FSPL losses are usually summed to calculate path losses [15]. Equation (7) describes the losses in free space, where the frequency (*f*) is expressed in MHz and the distance from the receiving antenna to the transmitting antenna *d* is given in kilometers [59]:

(7) PL(dB)=32.44+20logd+20logf

**Figure 9:**
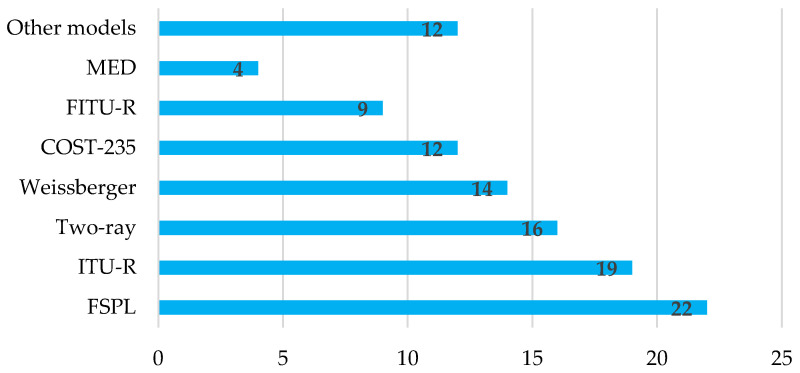
Most reported models in the SLR.

Figure 9 shows the most used models according to the articles that were subject to SLR according to the number of times they were considered in the different studies. Several papers considered more than one model in the characterization of loss in different scenarios or the comparative analysis of new proposals.

Although the models described in this section have already been validated and widely used, they generally present restrictions in their use, which have an impact on the efficiency of loss estimation Therefore, many studies focus on the development of new propagation models. For example, in Refs. [18,43,44,56,60,61] empirical models were proposed based on the estimation of signal power losses in each of the scenarios considered in the respective papers. As documented in the widely known models, deterministic proposals are not commonly used; however, some developments are also presented in Refs. [53,62]. Table 3 contains a summary of the most important characteristics of the models commonly used in research related to radio wave propagation in vegetated environments.

### 5.2. Application Scenarios for Propagation Studies

Most of the models presented in Section 5.1 are used in environments where the presence of vegetation affects the power of the radio signal. This SLR focuses on the study of WSN radio wave propagation models in agricultural and forestry fields. Many articles have estimated the effectiveness of new or validated models in scenarios where vegetation is present but which do not necessarily correspond to crop fields. Specifically, four types of environments predominate in the works reviewed: (1) agricultural fields, (2) agricultural greenhouses, (3) lawns or pastures, and (4) forest or jungle.

As for agricultural fields, also called outdoor crops, radio wave propagation has been modeled for a considerable number of crop types. The SLR highlights that most of the work is in cereal fields, especially rice [41,50,54,72,73], corn [25,41,74], and wheat [12,75,76]. Fruit plantations, which are also part of the outdoor crops category, have been mainly: mango [39,40,41] and guava [39,41].

Greenhouse crops, unlike traditional outdoor cultivation, require a closed circuit and protection from the outside, which directly affects the radio signal propagation conditions. There are not many papers presented in this SLR that consider this type of scenario in greenhouse contexts. Representative cases include the simulations developed in Ref. [29], where different models are evaluated without detailing the characteristics of the plantation. Greenhouse propagation studies of tomato seedlings were conducted in Refs. [18,47].

For grass or turf crop scenarios, most research estimates signal power loss in environments with grass of considerable height, as presented in Refs. [51,52,77,78]. Short-grass scenarios represent fewer challenges from the point of view of radio wave propagation; some of these analyses are presented in Refs. [16,77,79]. A special case is presented in Ref. [80], where the calculation of foliar losses is performed in sugarcane plantations, but, due to the characteristics of the plantation, it could also be considered as tall grass.

**Figure 10:**
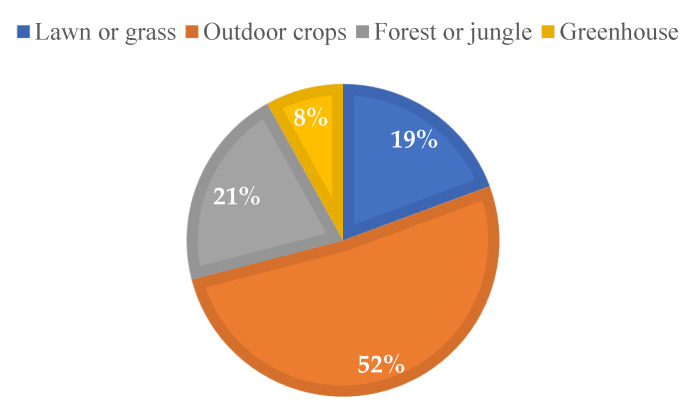
Distribution of application environments for propagation characterization work.

There are also results of work on the characterization of wireless signal attenuation in forests or jungles. In this case, most of the articles do not present detailed information on the type of shrubs present in these environments. Pine forests are the most referenced, presented in Refs. [62,81,82], as well as oak forests, also studied in Refs. [62,82]. In Refs. [43,52,56,60], it is indicated that the considered scenario corresponds to a forest, while, in Ref. [51], it was characterized as an environment of scattered trees. In this same category are classified the jungle environments, which are not extensively examined in this SLR since few studies were found on this aspect, such as Refs. [83,84]. Figure 10 shows the distribution of the environments that were considered in the articles that were part of this SLR.

### 5.3. Wireless Technologies, Frequency Bands, and Tools Used

Two technical factors are important in work conducted on radio wave propagation: (a) wireless technology used in data transmission and (b) frequency band. These two aspects are important in the selection of the technological tool to be used to perform *RSSI* measurements, which is the parameter used to determine the received power at the receiver. Equation (8) is used to estimate this value [48]:

(8) $$RSSI=10\log P_{r\left(d\right)}.\;$$

where *Pr*(*d*) corresponds to the received power and *d* is the distance from the transmitter to the receiver. In addition, the relationship between the power at the receiver and the path loss (PL) is expressed by Equation (9) [85]:

(9) $$P_{L\left(d\right)}=P_{t}+G_{t}+G_{r}-P_{r\left(d\right)}.\;$$

where *Pt* is the transmit power in dBm, *Gt* is the transmit antenna gain in dBi, *Gr* is the receiver antenna gain in dBi.

Different wireless technologies can be used for the transmission of data obtained in the motes that make up a WSN, and their choice is conditioned by different factors, including transmission speed, coverage, power consumption, and cost. Research on radio wave propagation in WSNs applied to the environments that have been the subject of analysis of this SLR indicates that the trend in wireless communication technology is towards the use of Zigbee, followed by the Long-Range Wide Area Network (LoRaWAN) protocol. In addition, there are reports of measurements with Bluetooth and LoRa. Table 4 presents a comparison of these standards. In addition, there are reports of measurements with WiFi and Bluetooth. Table 4 presents a comparison of these standards.

The technology most used in the research is Zigbee, a protocol based on the IEEE 802.15.4 standard. Some of its main features are low transmission speed, low power consumption, and low cost [86]. Of the spectrum usage possibilities offered by Zigbee, 2.4 GHz, which is part of the ISM (Industrial, Scientific, and Medical) band, was the most widely used in the research consulted in this SLR. In the development of new propagation models and the characterization of attenuation in the different environments presented in the previous section, most of the projects favored the use of commercial equipment. XBee devices were the most widely used in the development of new models, as presented in Refs. [38,41,42,44,46,50,52,53,61,74,75,83,90,91]. Texas Instruments transceivers were also widely used in those research studies that opted for Zigbee communication technology, being considered in the development of new models [18], or in the propagation characterization performed in Refs. [74,92]. Not all the research with Zigbee implementation worked in the 2.4 GHz band. For example, in Ref. [54], the operating frequency of the transceiver modules was 920 MHz.

Other articles report the use of WiFi in the implementation of the wireless solution. This technology is based on the IEEE 802.11 standard, and some of its features are high speed, low coverage, and reduced power consumption [93]. All the articles reporting their implementation used the 2.4 GHz band, with equipment working on the IEEE 802.11g standard [60,94], IEEE 802.11b [73,94], and IEEE 802.11n [94]. No information on the standard or working frequency was presented in Ref. [95], and no data on the equipment used was presented in Ref. [56].

Other technologies meet the conditions that allow their use in the communication stage of WSN motes, but their use is not widely reported in the articles analyzed by this SLR. Bluetooth, which is based on the IEEE 802.15.1 standard, is characterized by high speed, low cost, and reduced coverage. In this review, evidence of its use was only found in Ref. [83]. It was one of the wireless solutions used in the evaluation of technologies, operating the tests in the 2.4 GHz band.

LoRaWAN is another specification that can be used in WSN communications infrastructure in agricultural or similar environments. It is a wide-area, low-speed, high-coverage-range, and reduced-power-consumption protocol [96]. One of its main advantages is that it works in bands below 1 GHz [97], which is advantageous in signal propagation. In Ref. [98], the author does not develop a new model and does not characterize the attenuation in the environment, but this technology was used to calculate losses in the environment using propagation models. Although the number of papers reporting the use of LoRa is small compared to Zigbee, due to its characteristics, it is possibly the most promising wireless technology in WSN-based IoT applications in scenarios that require wide coverage, as is the case of cropping systems, the reason for which is its use increased considerably between 2020 and 2021, reported in Refs. [99,100,101,102].

**Figure 11:**
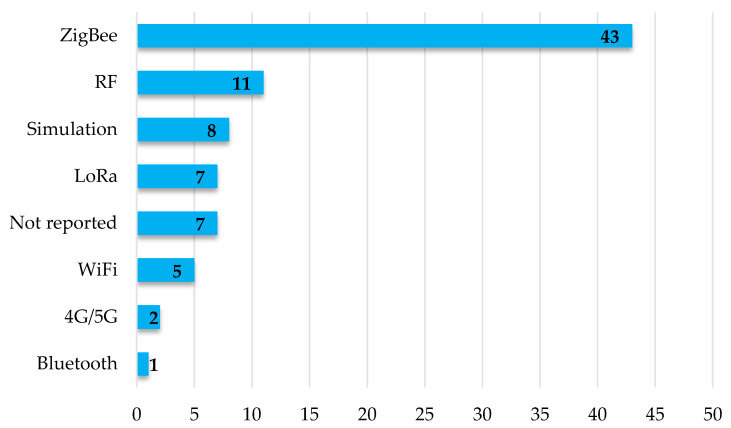
Number of articles that used the different communication technologies that appear in the SLR.

In the works that do not present a specific wireless communication standard and that are related to WSN planning, radiofrequency (RF) equipment (signal generators and spectrum analyzers) that are generally used in field tests were available. Although, in these circumstances, the use of the 2.4 GHz band is also predominant, and an additional set of frequencies of the radio spectrum is part of other works. In this type of study, the development of new propagation models was not a common activity, with only one report in Ref. [43], in which the selected frequency band was 433 MHz. The rest of the papers in which they used RF equipment at the time of *RSSI* measurements present attenuation characterization or model comparison activities. Within the set of frequencies evaluated in the use of such tools are the 3.5 GHz and 5.8 GHz frequencies examined in Ref. [82], the frequencies at 500 MHz, 1.2 GHz, and 2.5 GHz used in Ref. [40], while, in [41,43], they performed measurements at 870 MHz. Figure 11 presents information on the number of articles referring to each communication technology.

## 6. Discussion

We discuss the analysis of the techniques used in different investigations. We analyze the techniques used to determine the effectiveness in terms of attenuation prediction in the models used. It is important to note that there is no uniformity in the criteria used in the evaluation of the results, and, in many cases, there is no quantitative description. In contrast, much of the work that has considered factors other than frequency and distance has focused on loss behavior at different antenna heights or the type of foliage or distribution of trees in the environment. This section presents the most used evaluation methods during SLR.

This review identified the absence of standard validation techniques that determine the effectiveness of radio wave propagation models in agricultural or forestry environments. In most of the articles, the validation of the effectiveness of the propagation models was performed based on a comparative analysis of field measurements at different distances between the transmitter and receiver nodes. Some works do not make a conversion from *RSSI* to decibel losses [18,50,60,72,90,95]. In other cases [27,40,41], the analysis was conducted from the path loss contrast, considering the depth of the vegetation.

Regarding radio spectrum, although ISM bands facilitate the deployment of WSNs in PA, unlike licensed spectrum, excessive use of the 2.4 GHz portion increases the future probability of interference due to saturation of this band. Many measurements were taken in the 2.4 GHz band, and, at times, attenuation at other frequencies was also characterized. For example, in Ref. [40], they performed tests at 500 MHz; in Ref. [43], they took data at 870 MHz, while, in Ref. [54], it was completed at 920 MHz.

More than 15 validated propagation models are identified from this SLR. However, the results show that there is a preference both for the use of certain general models not developed for agricultural, forestry, or similar environments (e.g., FSPL and Two-Ray) and the use of vegetation models (e.g., MED, COST-235, FITU-R, ITU-R, and Weissberger). Therefore, the discussion of the results has been based on the models presented in Section 5.1, without suggesting that other models should not be considered. As for the criteria used to identify the level of effectiveness of the validated models, the most frequently used techniques are the root mean square error (*RMSE*), the mean absolute percentage error (*MAPE*), and the graphical method. Of these three, the most widely used method is the *RMSE*, calculated by Equation (10) [42]:

(10) $$RMSE=\sqrt{\frac{1}{n}*\overset{n}{\underset{i=1}{\sum }}{\left(x_{i}-{\hat{x}}_{i}\right)}^{2}}.\;$$

where *x_i_*, *y*, ${\hat{x}}_{i}$. are measured and predicted values, respectively, while *n* corresponds to the number of samples taken in the experiment.

Another statistical technique used is *MAPE*, which is less susceptible to extreme values [46] and is obtained by Equation (11):

(11) $$MAPE=\frac{1}{n_{k}}\overset{n_{k}}{\underset{i=1}{\sum }}\left|\frac{x_{i}-{\hat{x}}_{i}}{x_{i}}\right|\times 100\%.\;$$

where *x_i_* is the *i*-th empirical value, ${\hat{x}}_{i}$. is the *i*-th theoretical value, and *n_k_* is the number of samples. In both cases (*RMSE* and *MAPE*), the closer its value is to zero, the better the efficiency of the model [103].

Finally, many papers perform the analysis of the results from graphical means, making comparisons of distance regarding losses or *RSSI*. It is important to note that this type of analysis usually shows an underestimation or overestimation of losses without presenting quantitative information. Therefore, the results and conclusions presented could be subjective, which would reduce scientific rigor.

In the case of processes to evaluate the effectiveness of the proposed new models, the coefficient of determination, also called *R*^2^, is usually used, which is a widely used metric when applying regressions. In this case, the closer the *R*^2^ is to a value of one, the better the correlation with the real values [104]. The coefficient of determination is calculated using Equation (12) [46]:

(12) $$R^{2}=\frac{Explained\;Variation}{Total\;Variation}=\frac{\sum_{i=1}^{N}{\left(x_{i}-\bar{x}\right)}^{2}}{\sum_{i=1}^{N}{\left({\hat{x}}_{i}-\bar{x}\right)}^{2}}$$

Although the statistical techniques presented in this section have been widely used in the validation of different models, each of them has characteristics that make them more useful under certain conditions. This becomes an opportunity to develop validation techniques that apply to different models and under different test conditions. Table 5 summarizes the criteria used in the delimitation of the model analysis, consistent with the information provided earlier in this SLR.

## 7. Results

The results are divided into two approaches. First, the effectiveness of the models described in Section 5.1 is examined and, in the second part, the behavior of the new models is detailed. The results were analyzed in articles with information on their effectiveness in predicting signal loss, using quantitative data shown in tables, figures, or detailed text.

As for COST-235, only Ref. [41] reports similar predictions to measurements made on different outdoor crops. In Ref. [26], the authors state that the values obtained using this model were like their field measurements. However, they presented a loss overestimation of more than 10 dB difference. The other articles show that the performance of this model was deficient, presenting even very high errors, as in Ref. [46], underestimating losses with *MAPE* values up to 78%, or in Ref. [18], where the percentage error calculated in short distances was higher than 50%. Even when combined with FSPL and Two-Ray, the estimation of losses is far from the real measurements, as is the case presented in Ref. [53], which shows errors of 10.69% and 38.14% for the sum with each of the models.

The results obtained using the ITU-R demonstrate its inability to maintain a low level of error when describing signal loss in scenarios with the presence of vegetation, as well as the COST-235, preventing correct planning of WSN nodes. For example, in Refs. [38,67], they maintained a behavior close to the measurements, but, as the distance between the transmitter and receiver increased, they overestimated the measurements. Moreover, in Ref. [67], they used two antenna heights in two experimental scenarios; the first one places the antennas at 2.5 m and 3.4 m from the ground, presenting underestimation. The second scenario, with heights of 2.5 m and 4.4 m, fitted the measurements better. The remaining publications conclusively indicate that ITU-R is not able to characterize losses efficiently, confirming that variations in the environment and experimental conditions affect the results. Thus, in Ref. [40], the *RMSE* values range from 9.8 dB to 24.2 dB for different combinations of antenna heights and frequencies evaluated in mango plantations. On the other hand, under the same considerations in palm plantations, the *RMSE* values ranged from 4.9 dB to 18.1 dB. In this study, they combined the ITU-R model and the FSPL, achieving an average *RMSE* of 1.7 dB. For this model, there are reports of studies with high errors. For example, in the forest environment of Ref. [46], the MAPE values were 89%, and the *RMSE* reached 87.5 dB.

Regarding FITU-R, it only performed well in estimating losses under certain experimental configurations. In Ref. [38], the measurements and predictions are similar in leafless cherry crops, but, in the presence of foliage, there is an underestimation. In Ref. [62], this model is overestimated by reducing the height of the antennae near the canopy. In some cases, it was combined with other models not specifically developed to measure casing losses to increase its efficiency. For example, in Ref. [18], when combined with FSPL, the percentage error was less than 5% when the distance from the transmitter to the receiver was equal to or greater than 9.8 meters, but, at short distances, the error is high, reaching 35.22% at 2.6 meters. The rest of the articles analyzed show that the FITU-R is inadequate for predicting losses in the scenarios considered, although they do not report excessively high errors, unlike what occurred when other models (COST-235 and ITU-R) were used. In some scenarios, it presents underestimation when using the model [53], while, in Ref. [60], its prediction was above the measurements.

Regarding Weissberger, there is no information on the low levels of error in his estimation that establishes signal loss. Even with adjustments, as is the case presented in Ref. [42], the *R*^2^ only reaches a value of 0.67. The results of most papers show a tendency for this model to underestimate losses. Only in Ref. [18] were the FSPL and Two-Ray models combined, showing an overestimation in the measurements made inside a tomato greenhouse and an antenna height of 0.5 meters at its nodes. In Ref. [46], they recorded the highest error of this model, reaching a *MAPE* value of 39%.

Finally, concerning the vegetation models, the MED performed well in the application scenarios, although this does not indicate that it is suitable for sizing WSNs in agricultural or similar environments. In Ref. [40], they performed an adjustment process to improve its performance, obtaining *RMSE* values of 10.6 dB at 2.2 m and 2.57 dB at a measurement height of 2.6 meters. However, the band used was the 400 MHz band, which has much lower losses in contrast to other more commonly used bands, for example, the 2.4 GHz band.

The FSPL and Two-Ray models are widely used, either in comparison or in combination with other models. Regarding FSPL, all the articles report results that indicate significant differences when compared with measurements made in different study settings. In addition, there is no tendency to overestimate or underestimate. In Refs. [43,46,53], the solutions given by the model at each distance were below the obtained measurements. In contrast, in Ref. [56], he estimated above the real data. In all the articles analyzed, the change in the conditions in the experiment affected the results. For example, in Ref. [18], there was an underestimation when the antennas were located at 0.5 m, while, when increasing to 1.5 m, the behavior was of overestimation. Possibly due to its design characteristics, it presented high differences concerning the measurements. In Ref. [105], the minimum *MAPE* obtained was 30% at short distances, while Ref. [53] recorded values up to 82.44% using the same statistical analysis.

As for Two-Ray, there are also no case reports indicating an approximation to the empirical data. Its use generated overly high errors, especially in Ref. [18], where the percentage error was over 90% at any distance between the transmitter and receiver at an antenna height of 1.5 m, although, when reduced to 0.5 m, it decreased considerably to a maximum of 26.25%.

The compilation of results presented in the different works considered in this SLR demonstrates the low efficiency of traditional vegetation models when used as tools for loss estimation between the transmitter and receiver of a WSN. In addition, the lack of standardization in terms of the validation of the results and the limited information provided by some authors prevent a more detailed analysis of the results obtained in some experiments. However, the information gathered allows inferring that the vegetation models historically used in the characterization of attenuation in PA environments or with the presence of vegetation are not the most appropriate when used in the sizing of WSNs in these environments. Table 6 presents a summary of the references selected in the evaluation, with the results obtained in each case from the previously validated models.

As for the research aimed at developing new models considered in this review, the results are acceptable from a statistical point of view. In most cases, the criterion used was the *R*^2^, although there are reports of analysis of results based on *RMSE* and *MAPE*.

In three of the articles reviewed, the low estimates of the nontraditional models developed are reported. In Ref. [60], only 65.82% of the loss variations could be explained by the proposed regression model, a result that corresponds to the case where the node antenna is located at a height of 3.5 m since, at a height of 7 m, the *R*^2^ was 0.94. Ref. [50] also reports that the proposed development presents an acceptable behavior in the estimation of losses in a rice field during its different growth stages but does not present quantitative data. Finally, in Ref. [38], they indicate that the proposed model provides good agreement near the tree canopy but underestimates the attenuation at mid-height. Moreover, in this case, the error increases with the presence of leaves and with increasing distance. In the rest of the articles, the criteria used in the evaluation of the proposed models yielded favorable values, so it can be affirmed that they are capable of characterizing, from a statistical point of view, the attenuation in each of the environments studied. For example, in Refs. [78,105,106], the values of *R*^2^ were 0.95, 0.98, and 0.98, respectively. 

The *RMSE* was also used to validate the results of the new models. In Ref. [43], they used it in the validation phase of the effectiveness of their proposal concerning field measurements, obtaining values of 1.6 dB in the 870 MHz band and 1.5 dB in the 2.4 GHz band. In Ref. [62], they reported that the calculated *RMSE* was less than 1.5 dB for all the antenna heights and crop conditions considered. 

As for the relative error, only Ref. [48] reports its use as a statistic for the validation of the results. In this case, the proposed model had a difference of less than 2% concerning attenuation variations.

The results associated with the development of new models contrast with those obtained with traditional vegetation models. In this case, the models obtained from the measurements and the considerations of the specific environment in which the WSN is planned to be installed allow modeling losses with much lower error levels. However, the multiple variations of crops and associated environments represent a challenge for future researchers since it has not been possible to obtain a standard model that can be applied in all scenarios with good results and that avoids the characterization of each crop or wooded area where a WSN is intended to be installed. Table 7 presents a summary of the new models and their results.

## 8. Conclusions

The results of this SLR show that, although there is a large set of radio wave propagation models validated in vegetated environments, the characterization of attenuation in agricultural, or similar, environments is an area of engineering that requires further research. In addition, this SLR identifies the models most used in planning the sizing of WSNs in crops, as well as the scenarios in which most research has been conducted, as well as trends in the use of wireless technologies required for node connectivity. The criteria and values that determine the validity of a model and/or its effectiveness were also identified.

The number of articles in this research area had been increasing from 2015 to 2019, decreasing from 2020, probably a product of the SARS-CoV-2 pandemic, which forced the suspension of activities in different research areas. Although the number of articles found in publications not categorized as proceedings is high (50%), it is also important to note that 26% of the papers consulted in this SLR are found in journals or proceedings of events classified as Q1, which demonstrates the relevance of the research area for journals that address this type of topic.

Six countries account for most of the publications generated (China, India, Japan, Malaysia, Spain, and the United States). The remaining articles come from 18 other nations, demonstrating that there is a diversity of institutions around the world researching this topic.

The technical analysis allows us to identify that, of the validated and recognized models, the most widely used are of the empirical type, highlighting the use of the MED and its derivatives. However, the results of *RMSE* above 10 dB and *MAPE* above 10% in most investigations show that vegetation models are inefficient in estimating radio signal loss in foliage when applied in scenarios other than those considered in their development. Therefore, adjustments are needed to increase the accuracy under specific experimental conditions and in the configuration of the communication system elements.

Several papers consider non-specific models for vegetated environments (e.g., FSPL and Two-Ray). However, these are generally used as an aid to calculate attenuation over the entire communication link and not only in vegetation. Like vegetation models, they lead to high errors. The results reported on the use of vegetation models and their effectiveness in estimating attenuation in the scenarios studied indicate that none can be rated as the most accurate in predicting signal losses in environments with the presence of bushes, plants, grass, or similar. All the vegetation models considered showed variations in their accuracy in the cases in which they were applied, with no definite trend of estimation above or below the real loss values. The poor effectiveness of vegetation models demonstrates the need for more comprehensive developments that include factors other than distance and frequency, e.g., antenna height, obstacle dimensions, and shrub spacing. 

As for the models considered new, most of them adequately fitted the attenuation curve in the scenarios in which they were developed, and, in the conditions proposed in different experiments, reached *R*^2^ values higher than 0.9 in most of the investigations.

Regarding the scenarios considered in the characterization of attenuation and the evaluation or development of new models, most of the studies focus on outdoor agricultural environments and greenhouses. In these, since there is generally a uniform layout and one type of shrub predominates in the fields, modeling the effects of vegetation on the signal in the wireless channel is facilitated. As for environments dominated by grasses or pastures (common in livestock environments), they present favorable conditions for radio wave propagation. However, the results show that, in most cases, it is necessary to adjust the vegetation models to reduce error levels. As for forest environments, which, in many cases, have similarities with some types of crops, they represent a great challenge in the estimation of signal loss because the uniform positioning of trees and plants does not predominate. In addition, it is normal that there are different species of vegetation, and each of them has its geometrical characteristics.

Regarding the wireless technologies used in WSNs for agricultural applications, the results show that, from 2011 to 2021, there is a preference for the use of Zigbee. However, as of 2019, there is a growing trend in the use of LoRa. Its coverage, power consumption, and cost characteristics position it as one of the wireless technologies with the highest future demand in WSN and IoT applications. 

## 9. Future Works

Future developments of new models should further study the effects of plant geometry, composition, and layout on radio signal loss. In the case of greenhouse crops, it is desirable to study how the infrastructure that houses them influences signal attenuation. For the study of radio wave propagation in agricultural environments or with the presence of bushes or grass, it is advisable to use techniques that have not been used in studies of radio wave propagation in these scenarios, such as machine learning. In addition, this SLR identifies the lack of standard validation techniques to adequately evaluate and compare propagation models centered on PA scenarios, opening the possibility of lines of research in this area. It is also suggested to analyze those technologies that have not been widely documented and that have interesting features, such as large coverage, e.g., Sigfox.

Regarding the application context, studies are proposed focused on crops of great importance for food sustainability, such as cassava, banana, or soybean, and ecosystems located in marine–coastal transition zones, such as mangrove forests, among others.

## Figures and Tables

**Table 1:** Thematic axes and combination strategies used in the review.

Thematic Axis 1	Thematic Axis 2	Thematic Axis 3	Exclusion Criteria
Agriculture	WSN	Path loss	Algorithm
Crop		Path loss model	Image
Farm		Wave propagation	Indoor
Vegetation			Irrigation
			Localization
			Monitoring
			Solar energy
**Combinations**			
**Link 1:**	Axis 1 AND Axis 2		
**Link 2:**	Axis 1 AND Axis 2 AND NOT Exclusion criteria		
**Link 3:**	Axis 1 AND Axis 2 AND Axis 3		
**Link 4:**	Axis 1 AND Axis 2 AND Axis 3 NOT Exclusion criteria		
**Link 5:**	Axis 2 AND Axis 3		
**Link 6:**	Axis 2 AND Axis 3 NOT Exclusion criteria		

**Table 2:** Journals with the most publications.

Publishing	Number of Posts	Country	Type	ISSN	Quartile in SJR	Quartile in JCR
Computers and Electronics in Agriculture	9	Netherlands	Journal	1681699	Q1	Q1
Sensors	5	Switzerland	Journal	14243210	Q2	Q1
Progress in Electromagnetics Research C	3	USA	Journal	19378718	Q3	Q3
Wireless Personal Communications	3	Netherlands	Journal	1572834X	Q3	Q4

**Table 3:** Summary of propagation models most used in the SLR articles.

Model	Vegetative Model	Antenna Height	Antenna Gain	Conditions	Observations	References
MED	Yes	No	No	*d <* 400 m 200 MHz *< f* < 95 GHz	Applicable in communication links obstructed by dense, dry, leafy trees; present in temperate latitude forests.	[27,38,39,40,63]
ITU-R	Yes	No	No	*d* < 400 m 200 MHz < *f* < 95 GHz For f above 1 GHz, it only considers the diffracted components around the vegetation and from above, in addition to the component reflected from the ground.	It is proposed for cases where the transmitting or receiving antenna is located near a small grove of trees, allowing most of the signal to propagate through the vegetation.	[3,12,27,40,41,42,46,53,64,65,66]
COST-235	Yes	No	No	200 MHz < *f* < 95 GHz	Consider the presence and absence of leaves on trees. It can be used in vegetation scenarios up to 200 meters wide.	[3,12,18,26,41,46,53,60,67,68]
FITU-R	Yes	No	No	*d* < 400 m	Consider the presence and absence of leaves on trees. Suitable for modeling the propagation of radio waves in different seasons of the year.	[3,9,53,60,63]
Weissberger	Yes	No	No	*d* < 400 m 230 MHz < *f* < 95 GHz	Useful when the path between the transmitter and the receiver is occupied by dense vegetation consisting of trees with low humidity.	[3,12,18,26,40,41,42,46,53,64]
FSPL	No	No	Yes	*d* >> *λ*	This model does not consider the mechanisms of radio wave propagation (e.g., reflection, diffraction, refraction, absorption). It is used as a reference to compare the performance of different wireless communication technologies. It is also a complement to vegetation models to calculate losses over the entire channel path.	[15,16,26,39,46,48,56,57,69,70,71]
Two-Ray	No	Yes	Yes	*d* >> *ht* + *hr*	This model considers the effects of the ground and the reflection of the ray LOS (line-of-sight). Useful for modeling propagation over long distances. It is also a complement to vegetation models to calculate losses over the entire channel path.	[15,16,45,46,48,69]

**Table 4:** Comparison of Bluetooth, LoRaWAN, WiFi, and Zigbee technologies. Sources: Refs. [86,87,88,89].

Technology	Frequency Bands	Speed Rate	Coverage Range (Typical)	Energy Consumption
Bluetooth	2.4 GHz	720 kbps–1 Mbps	1–10 m	Low
LoRaWAN	433 MHz, 868 MHz	250 bps–50 kbps	>10 km	Low
WiFi	2.4 GHz, 5 GHz	1.2 Mbps–54 Mbps	Hasta 100 m	High
Zigbee	870 MHz, 902–928 MHz, 2.4 GHz	20 kbps–250 kbps	10 m–1.6 km	Low

**Table 5:** Criteria used in SLR performance evaluation.

Canon	Measure
Models	COST-235, ITU-R, FITU-R, Weissberger, MED, FSPL, Two-ray
Characterization of attenuation	Loss vs. distance *RSSI* vs. distance
Track predictions	Graphical method *RMSE* *MAPE* *R*^2^
Prediction effectiveness	Follow measurements closely Overestimate Underestimate

**Table 6:** Summary of the results of the evaluation of the previously validated models.

Reference	Models	Environment	Technology	Summary of Results
[16]	FSPL, Two-Ray	Grass	RF equipment	- Two-Ray and FSPL do not adequately represent measurements.
[12]	COST-235, ITU-R, Weissberger	Agricultural fields	Zigbee	- COST-235 was the largest overvaluation of losses in all growth stages of the crop. - Weissberger was the one with the greatest underestimation of losses in all stages of the crop.
[27]	COST-235, FSPL, Weissberger	Agricultural fields	RF equipment	- FSPL, Weissberger, and ITU-R show similar trends and differ from the observed path loss values. - COST 235 features higher precision.
[38]	ITU-R	Agricultural fields	Zigbee	- FITU-R was the most accurate in a setting with no leaves on the trees. - FSPL presented the highest difference concerning the measures.
[40]	FITU-R, ITU-R, Weissberger	Agricultural fields	RF equipment	- FITU-R was more accurate at frequencies below 2 GHz. - COST235 was more accurate at frequencies above 2 GHz.
[39]	FSPL, MED	Agricultural fields	Zigbee	- FSPL, adjusted to the measurements, was the most accurate with grass heights of 15 cm (*RMSE* of 4.5) and 1 m (1.62). - MED, adjusted to measurements, was the least accurate with grass heights of 15 cm (*RMSE* of 4.65) and 1 m (1.9).
[42]	ITU-R, Weissberger	Agricultural fields	Zigbee	- Weissberger was the most accurate with an R2 of 0.62 when adjusted.
[43]	COST-235, FITU-R, FSPL, ITU-R	Agricultural fields	RF equipment	- FITU-R was more accurate at 2.4 GHz. - ITU-R and FITU-R show similar trends at 870 MHz. - FSPL was the one with the greatest difference concerning the measurements.
[44]	COST-235, FITU-R, Weissberger	Agricultural fields	Zigbee	- FITU-R, in all the combinations of the experiment, presented the highest precision (*RMSE* between 3.8 and 13.6). - COST-235, in all the combinations of the experiment, presented the highest overvaluation (*RMSE* between 8.0 and 26.8).
[18]	COST-235, FSPL, ITU-R, Two-Ray, Weissberger	Greenhouse	Zigbee	- Two-Ray presented was more accurate with node antenna height of 0.5 m (% error between 0.65% and 26.25%). - FSPL presented the greatest difference with node antenna height of 0.5 m (% error between 4.52% and 42.84%). - ITU-R added with Two-Ray and Weissberger added with Two-Ray were the most accurate with a node antenna height of 1.5 m.
[46]	COST-235, FSPL, ITU-R, Two-Ray, Weissberger	Forest or jungle	Zigbee	- Two-Ray was more accurate (*MAPE* 25%). - ITU-R was the one with the greatest difference concerning the measurements (*MAPE* of 89%).
[48]	FSPL, Two-Ray	Agricultural fields	Zigbee	- FSPL presented a low performance in all stages of the culture (*RMSE* between 10.33 dB and 18.19 dB). - Two-Ray showed a decrease in *RMSE* when increasing the height of the node antenna.
[53]	COST-235, FITU-R, FSPL, ITU-R, Two-Ray, Weissberger	Greenhouse	Zigbee	- COST-235 added with FSPL was the most accurate (*MAPE* of 10.69%). - Weissberger added with Two-Ray presented the greatest difference concerning the measurements.
[56]	FSPL	Forest or jungle	WiFi	- FSPL presented high overvaluation of the measurements.
[58]	COST-235, FITU-R, FSPL, Weissberger	Forest or jungle	RF equipment	- All models underestimated the measurements (*RMSE* up to 68 dB at 25 m).
[60]	COST-235, FITU-R	Forest or jungle	WiFi	- FITU-R and COST-235 presented high overvaluation of the measurements.
[82]	FSPL, ITU-R	Forest or jungle	RF equipment	- ITU-R overestimates at distances less than 20 m and underestimates at distances greater than 20 m. - FSPL overestimates measurements.
[67]	COST-235, FITU-R, ITU-R, MED	Agricultural fields	Zigbee	- MED was more accurate with antenna heights of 2.0 m and 2.3 m. - ITU-R had good prediction in short distances (up to 54 m) with an antenna height of 2.3 m. - FITU-R improved performance as distance increased with 2.0 m and 2.3 m antenna height. - COST-235 overestimated measurements in all cases.
[104]	FSPL, Two-Ray	Forest and grass	Zigbee	- Two-Ray was the most accurate (average *RMSE* of 19.8). - FSPL presented the highest difference concerning the measurements (mean *RMSE* of 34.37).
[106]	FSPL, ITU-R, Two-Ray	Agricultural fields	Zigbee	- Without adjusting, Two-Ray was more accurate with an antenna height of 0.5 m (average *RMSE* of 10.33 dB). - Without adjusting, FSPL was more accurate with antenna heights of 1.0 m and 1.5 m (average *RMSE* of 10.22 dB and 8.26 dB, respectively). - Without adjusting, ITU-R had the highest difference concerning the measurements in all scenarios (*RMSE* greater than 60 dB). - By adjusting Two-Ray, it was the most accurate in all scenarios (average *RMSE* less than 2.5 dB). - When adjusting, it presented a greater difference with the measurements (average *RMSE* between 1.93 dB to 2.06 dB).

**Table 7:** Summary of evaluation results of new models.

Reference	Environment	Technology	Summary of Results
[43]	Forest or jungle	RF equipment	- *RMSE* = 1.6 dB at 870 MHz. - *RMSE* = 1.5 dB at 2.4 GHz.
[44]	Agricultural field	Zigbee	- Average *RMSE* of 4.48 in all combinations of the experiment. - *R*^2^ between 0.87 and 0.9 with logarithmic adjustment.
[48]	Agricultural field	Zigbee	- Relative error < 2%
[60]	Forest or jungle	WiFi	- *R*^2^ = 0.66 with antenna height of 3.5 m. - *R*^2^ = 0.94 with antenna height of 7 m.
[38]	Agricultural field	Zigbee	- Good match near the treetops. - Underestimation at a medium height. - The error increased with the presence of leaves and with increasing distance.
[18]	Greenhouse	Zigbee	- % average error = 2.76% with a minimum of 0.06% and a maximum of 8.81% at distances of 2.6 m and 13.4 m, respectively, with antenna height of 0.5 m. - % average error = 1.57% with a minimum of 0.26% and a maximum of 3.01% at distances of 6.2 m and 4.4 m, respectively, with antenna height of 1.5 m.
[50]	Agricultural field	Zigbee	- Acceptable results.
[62]	Forest or jungle	RF equipment	- *RMSE* between 0.97 to 1.49 with antenna height of 2 m. - *RMSE* 0.19 to 1.33 with 3 m antenna height.
[77]	Grass	Zigbee	- *R*^2^ = 0.95 in short grass. - *R*^2^ = 0.92 in tall grass.
[104]	Forestry and turf	Zigbee	- *R*^2^ = 0.99 in grass. - *R*^2^ = 1 in a sparse tree environment.
[105]	Forest or jungle	Zigbee	- *R*^2^ = 0.98
[106]	Agricultural field	RF equipment	- *R*^2^ = 0.98 under (LoS) conditions. - *R*^2^ = 0.98 under non-line-of-sight (NLoS) conditions.
